# Voluntary Deep Inspiration Breath-Hold (VDIBH) Whole-Breast Irradiation Assisted by Optical Surface Monitoring System (OSMS) in Patients With Left-Sided Breast Cancer: A Prospective Phase II Study

**DOI:** 10.1177/15330338231173773

**Published:** 2023-06-14

**Authors:** Jiang-Hu Zhang, Tan-Tan Li, Shi-Rui Qin, Zhi-Qiang Liu, Si-Ye Chen, Yong-Wen Song, Yu Tang, Hao Jing, Hui Fang, Xu-Ran Zhao, Jing Jin, Yue-Ping Liu, Yuan Tang, Shu-Nan Qi, Ning Li, Bo Chen, Ning-Ning Lu, Ye-Xiong Li, Shu-Lian Wang

**Affiliations:** 1 National Cancer Center/National Clinical Research Center for Cancer/Cancer Hospital, 12501 Chinese Academy of Medical Sciences and Peking Union Medical College, Beijing, China

**Keywords:** breast cancer, whole breast irradiation, voluntary deep inspiration breath-hold, optical surface monitoring system, cardiopulmonary dose

## Abstract

**Objectives:** To investigate the dosimetric advantages of the voluntary deep inspiration breath-hold technique assisted by optical surface monitoring system for whole breast irradiation in left breast cancer after breast-conserving surgery and verify the reproducibility and acceptability of this technique. **Methods:** Twenty patients with left breast cancer receiving whole breast irradiation after breast-conserving surgery were enrolled in this prospective phase II study. Computed tomography simulation was performed during both free breathing and voluntary deep inspiration breath-hold for all patients. Whole breast irradiation plans were designed, and the volumes and doses of the heart, left anterior descending coronary artery, and lung were compared between free breathing and voluntary deep inspiration breath-hold. Cone beam computed tomography was performed for the first 3 treatments, then weekly during voluntary deep inspiration breath-hold treatment to evaluate the accuracy of the optical surface monitoring system technique. The acceptance of this technique was evaluated with in-house questionnaires completed by patients and radiotherapists. **Results:** The median age was 45 (27-63) years. All patients received hypofractionated whole breast irradiation using intensity-modulated radiation therapy up to a total dose of 43.5 Gy/2.9 Gy/15f. Seventeen of the 20 patients received concomitant tumor bed boost to a total dose of 49.5 Gy/3.3 Gy/15f. Voluntary deep inspiration breath-hold showed a significant decrease in the heart mean dose (262 ± 163 cGy vs 515 ± 216 cGy, *P* < .001) and left anterior descending coronary artery (1191 ± 827 cGy vs 1794 ± 833 cGy, *P* < .001). The median delivery time of radiotherapy was 4 (1.5-11) min. The median deep breathing cycles were 4 (2-9) times. The average score for acceptance of voluntary deep inspiration breath-hold by patients and radiotherapists was 8.7 ± 0.9 (out of 12) and 10.6 ± 3.2 (out of 15), respectively, indicating good acceptance by both. **Conclusions:** The voluntary deep inspiration breath-hold technique for whole breast irradiation after breast-conserving surgery in patients with left breast cancer significantly reduces the cardiopulmonary dose. Optical surface monitoring system–assisted voluntary deep inspiration breath-hold is reproducible and feasible and showed good acceptance by both patients and radiotherapists.

## Introduction

Breast-conserving surgery (BCS) combined with radiotherapy (RT) has become the standard treatment for early breast cancer. Radiotherapy can significantly improve the local control rate and survival rate while conserving the breast. A 3.5-week schedule of hypofractionated RT is noninferior to a standard 6-week schedule of conventional fractionated RT in China, according to Wang *et al*.^1^ However, RT potentially increases the risk of cardiac toxicity owing to heart exposure, especially for left-sided breast cancer.^2,3^ The 15-year incidence of cardiovascular events in patients with left breast cancer treated with RT after BCS was 7.6%, compared with 1.1% for those without RT.^4^ The heart exposure dose was found to be an important predictor for cardiovascular events. Darby *et al* reported that the relative risk of major coronary events increased by 7.4% if the mean heart dose increased by 1 Gy.^5^

Compared to free-breathing (FB), deep-inspiration breath hold (DIBH) technique, by expanding the lung and increasing the distance between the heart and the chest wall, can effectively reduce the heart dose.^6–10^ However, repeated DIBHs bring intra- and interfraction uncertainties. For example, different breathing maneuvers (abdominal vs thoracic breathing) can lead to variations in breast position despite the same inspiration volume. To guarantee a reproducible position of both external target breast and internal organ-at-risk heart, image-guided RT during treatment is necessary. Cone beam computed tomography (CBCT) is frequently used to verify the setup accuracy and the level of deep inspiration but at the cost of additional patient exposure to non-therapeutic radiation dose. Optical surface tracking, a nonionizing indirect approach for real-time respiratory motion monitoring, has a stability and localization accuracy within 0.2 mm by tracking an infrared marker affixed over the patient's xiphoid process during DIBH.^11^ Optical surface monitoring system (OSMS) (VisionRT) projects visible light on the patient and detects the surface and surface movement caused by respiration. This movement detection can be used to verify the 3-dimensional breast surface position to gate the beam during treatment.^12–14^ Theoretically, it is a useful tool for the surveillance of DIBH treatment in breast cancer. Alderliesten *et al* reported good agreement in translational setup errors between CBCT and AlignRT based on a region of interest (ROI) incorporating the left-sided breast surface.^15^

Therefore, we conducted a prospective phase II study to evaluate the efficacy of voluntary deep inspiration breath-hold (VDIBH) on cardiopulmonary sparing and explored the feasibility of real-time image-guided RT by daily OSMS combined with less frequent CBCT.

## Materials and Methods

### Inclusion Criteria

This study (ClinicalTrials.gov, number NCT03461588) was investigator-initiated and designed as a prospective phase II trial. This study followed the Strengthening the Reporting of Observational Studies in Epidemiology (STROBE) reporting guideline.^16^ Between January 2017 and August 2017, we screened 25 patients and ultimately ruled out 5 patients, 3 patients who were unable to hold their breath for 35 s, 2 patients who were unable to cooperate with radiotherapists instructions. Finally, 20 consecutive women who met the following criteria were included: age between 18 and 70 years, confirmed left-sided breast cancer, the intention to receive whole breast irradiation (WBI, nodal irradiation was not performed) after BCS, proficiency in DIBH, ability to hold breath for at least 35 s and repeat for at least 6 cycles. We have de-identified patient details such that the identity of any person may not be ascertained in any way.

### Computed Tomography Simulation

**Figure 1.**
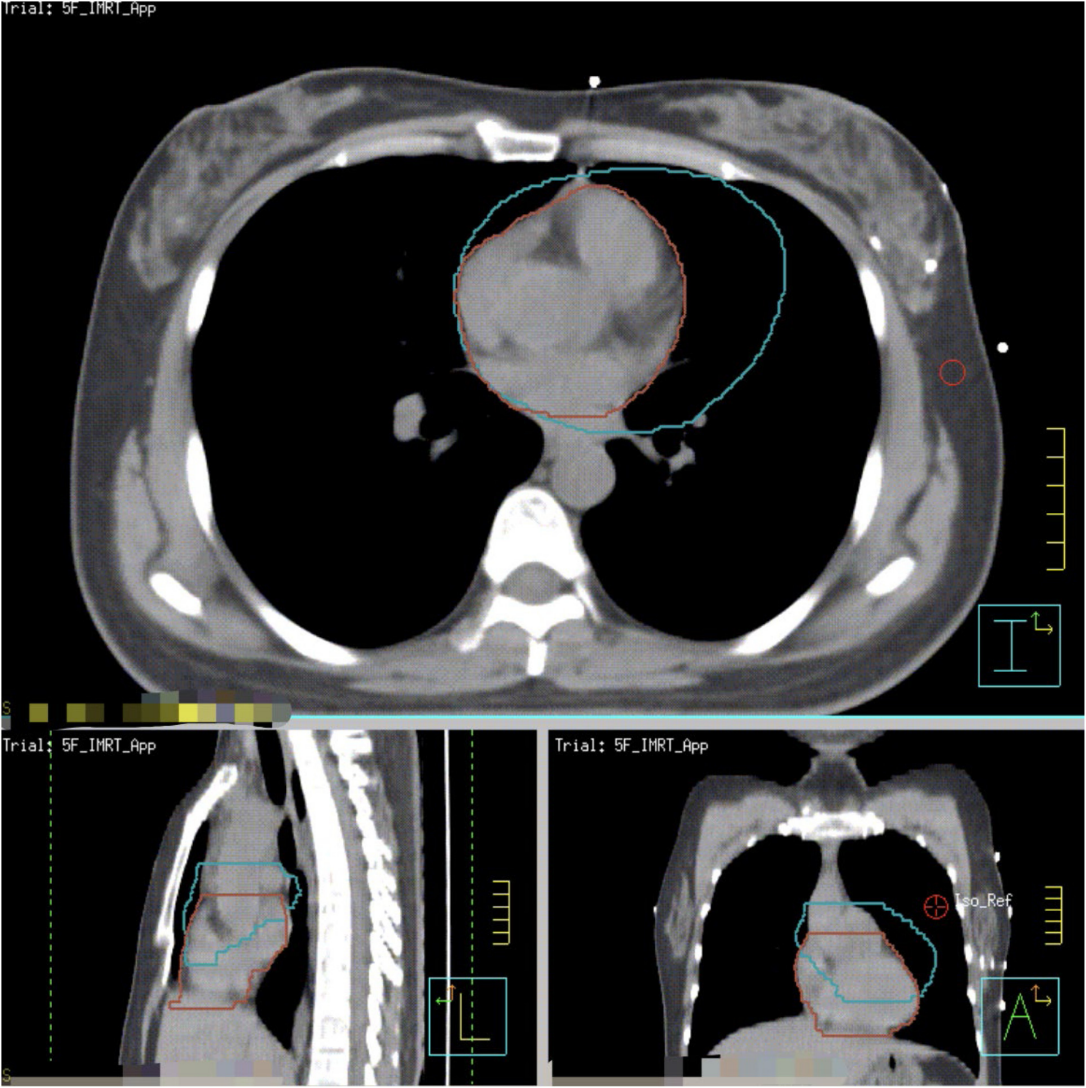
The heart displacement on voluntary deep inspiration breath-hold (VDIBH) as compared with free-breathing (FB) simulation. Green contour: heart position on FB; Brown contour: heart position on VDIBH.

All patients underwent CT simulation in a supine position immobilized on the breast bracket (CIVCO, Medical Solutions). Before simulation, preparatory DIBH coaching and training were performed with computer-controlled, visual feedback active breathing control (ABC) system (Elekta), and the threshold of inspiration volume was identified individually at a comfortable level, typically 70% to 90% of the maximum inspiration volume. Two sets of simulation CT scans, an FB CT scan, and an ABC assisted-DIBH CT scan using the identified threshold of inspiration volume from training were acquired using the same isocenter for each patient (Figure 1).

### Treatment Planning

Treatment plans were created on DIBH CTs. The FB and DIBH CTs are fused based on the left breast. The doses of the heart, left anterior descending coronary artery (LADCA), and left lung in FB and DIBH were evaluated according to the DIBH treatment plans for dosimetric comparison. Tangential-based field-in-field simplified intensity-modulated RT was used for these patients. The prescribed dose is 43.5 Gy in 15 daily fractions to the whole breast and 49.5 Gy in 15 daily fractions to the tumor bed if indicated. The DIBH technique was used for all treatments.

### Optical Surface Monitoring System Preparation

The DICOM data of the planning CT with skin contour and the isocenter coordinate were sent to the AlignRT workstation. A ROI of the external skin of the treatment area (unstable regions such as axillar were avoided) was selected to serve as the setup reference image. The tolerances were determined for real-time deltas of the on-treatment breath-hold ROI surface to the planned surface as 6-dimensional translation and rotation errors of 3 mm and 3°.

### Treatment Delivery

At the treatment machine, patients are set up under OSMS. The AlignRT system automatically acquires the ROI of the patient surface and registers it to the reference to calculate real-time deltas. The set-up is considered acceptable if the real-time deltas of the current breath-hold ROI surface to the planned surface are within predetermined tolerances. Cone beam computed tomography is performed to verify the internal target matches. During treatment, the patient is instructed to take a deep breath and hold it, and the radiation beam is turned on automatically while the patient is holding their breath and real-time deltas within predetermined tolerances (6-dimensional translation and rotation errors of 3 mm and 3°). Cone beam computed tomography is performed at least for the first 3 fractions, then once a week.

### Statistical Analyses

**Figure 2.**
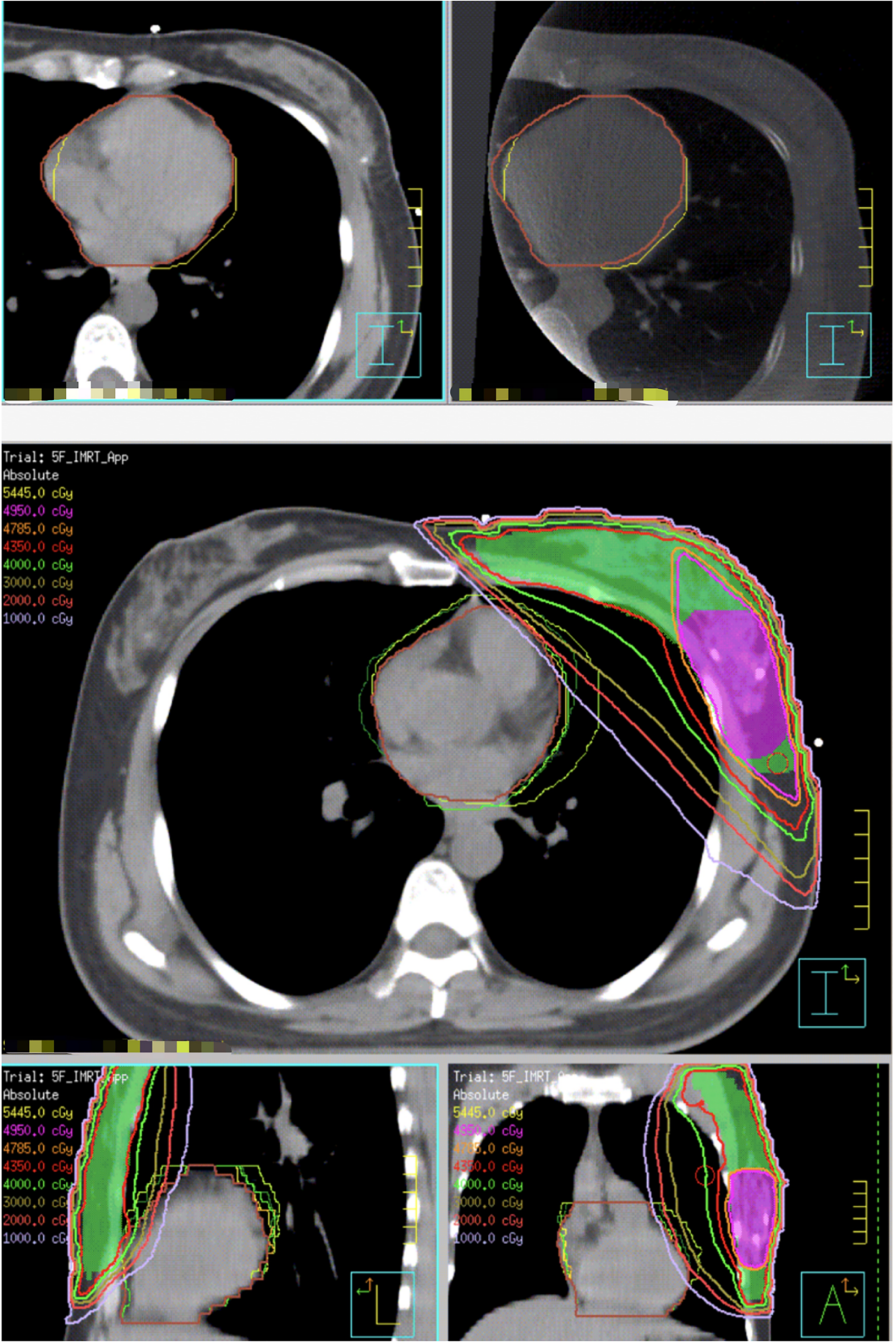
(A, upper left) Heart contour on the voluntary deep inspiration breath-hold (VDIBH)-planned computed tomography (CT) (brown); (B, upper right) heart contour on cone beam computed tomography (CBCT) (yellow); (C, lower middle) all heart contours from CBCT images registered to the voluntary deep inspiration breath-hold (VDIBH)-planned CT of one patient for heart position and heart dose evaluation.

All CBCT images were analyzed to assess whether the heart was displaced away from the chest in a manner consistent with the DIBH-planned images. The CBCT images were uploaded to the planning system and registered with the planning CTs based on the breast target. The heart during treatment was contoured on the CBCT (Figure 2). The heart position during treatment was evaluated based on the Dice similarity coefficient, DSC = 2* (CBCT∩DIBH-planned)/(CBCT∪DIBH-planned). In addition, the heart dose during treatment was estimated based on the initial DIBH treatment plan. The treatment delivery times of each fraction, recorded by the Mosaiq (Elekta), from initiating the first to finishing the last field of the treatment, were analyzed for all patients.

A predesigned questionnaire was completed by patients and involved radiotherapists to assess the acceptance of this technique. The translational set up errors were calculated by comparing CBCT with planning CT, and the planning target volume margins were calculated using the following formula: 2.5Σ + 0.7δ, wherein Σ and δ represent the standard deviations of the systematic and random errors, respectively.^17,18^ The data were statistically analyzed using SPSS version 25.0 (IBM Corporation), and measurement data were compared using the Student *t* test (normal distribution) or the Wilcoxon rank sum test (non-normal distribution), which is a nonparametric test. The test level was α = .05. Informed consent was obtained from all individual participants included in the study.

## Results

### Basic Characteristics of the 20 Participants

Twenty patients with left-sided breast cancer were included in this study. The median age was 45 years (27-63 years old). Three (15%), 10 (50%), and 6 (30%) patients had stage 0, I, and II disease, respectively. Seventeen patients (85%) received tumor bed boost and 3 (15%) patients did not. All patients completed the planned treatment without interruption.

### Dosimetric Comparison Between VDIBH and FB

The heart Dmean, LADCA, the total volume, and V20 of the left lung on FB and VDIBH are shown in Table 1. Voluntary deep inspiration breath-hold had significantly larger lung volume and lower doses to the heart, LADCA, and left lung than FB.

**Table 1.** The Dosimetric Comparison of Heart, LADCA, and Left Lung Between FB and VDIBH.

	FB	VDIBH	*P*
Heart mean dose (cGy)	515.1 (414-616.2)	262 (185.6-338.5)	<.001
LADCA mean dose (cGy)	1794.1 (1404.1-2184.2)	1192 (804.7-1579.3)	<.001
Left lung volume (mL)	1294 (1191-1396)	2037 (1810-2263)	<.001
Left lung V20 (%)	20.7 (18.8-22.6)	15.5 (14-17)	<.001

Abbreviations: VDIBH, voluntary deep inspiration breath-hold; FB, free breathing; LADCA, left anterior descending coronary artery.

**Figure 3.**
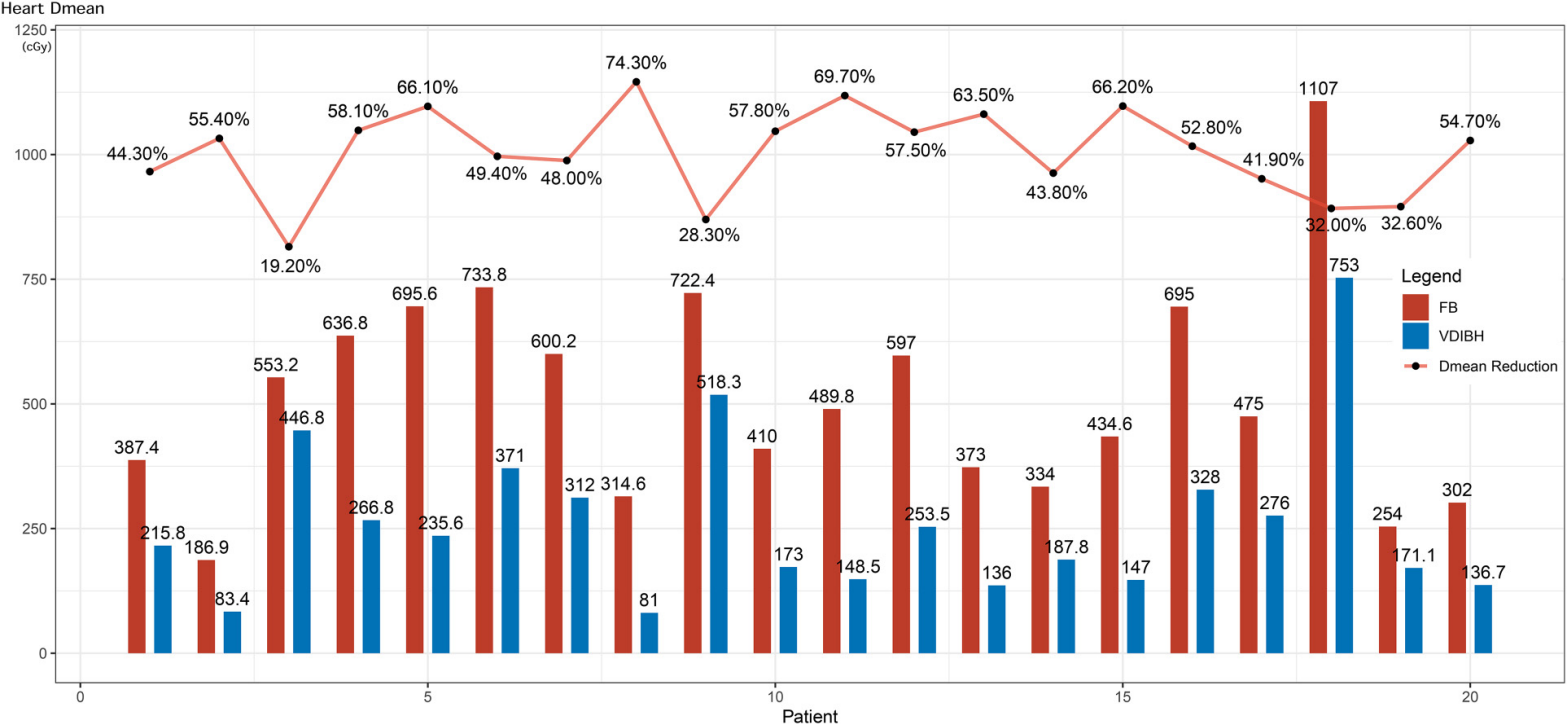
The heart D_mean_ of free breathing (FB) versus voluntary deep inspiration breath-hold (VDIBH), and the percentage of dose reduction by VDIBH for 20 patients.

Figure 3 shows the mean heart dose of FB versus VDIBH, and the percentage of dose reduction by VDIBH for the 20 individual patients. The heart D_mean_ reduction by VDIBH varied from 19.2% to 74.3%, with a median of 55.3%.

### Heart Position and Radiation Dose in Actual Treatment

**Figure 4.**
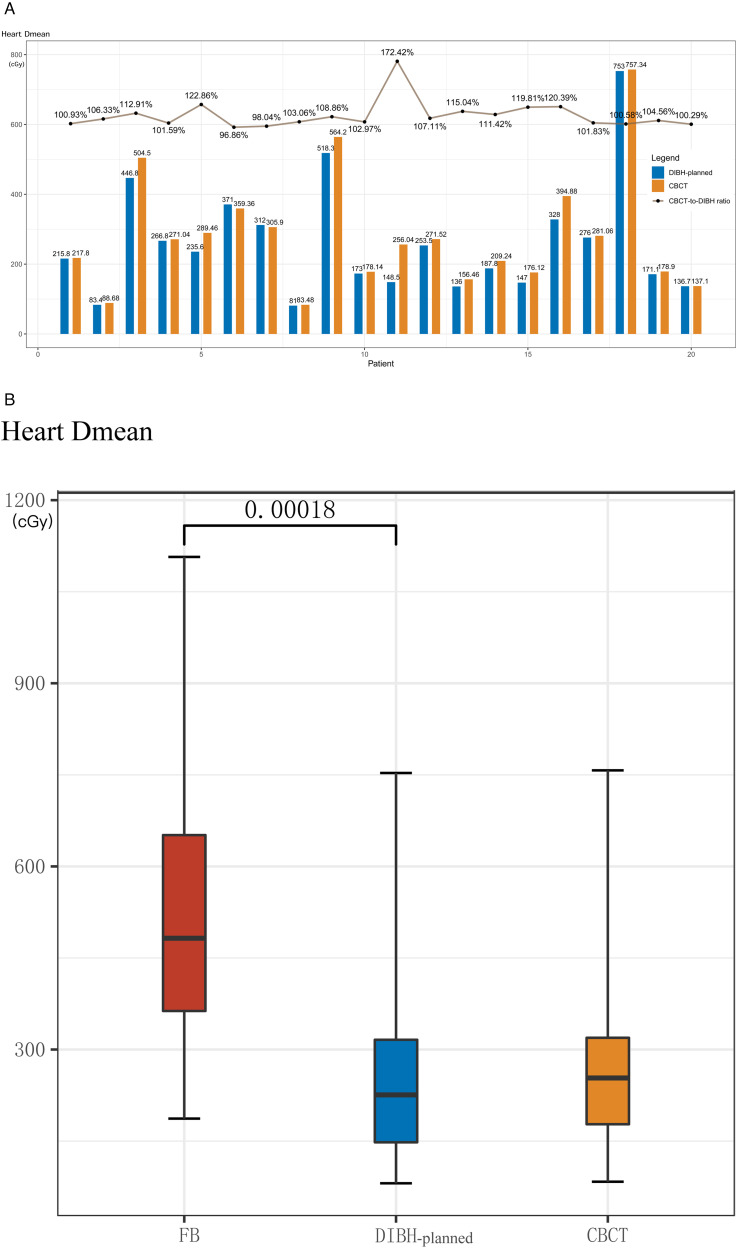
The comparison of heart D_mean_ among free breathing (FB), deep-inspiration breath hold (DIBH)-planned, and voluntary deep inspiration breath-hold (VDIBH) treatment (cone beam computed tomography [CBCT]). The ratio of heart D_mean_ of CBCT/DIBH-planned varied with a median of 105% (A). The heart D_mean_ of patients under VDIBH both in CBCT and DIBH-planned was significantly lower than that under FB (B).

A total of 98 CBCT images were uploaded to the planning system and registered with the planning CTs based on the breast target. The average dice similarity coefficient of heart contour from CBCT and DIBH-planned was 0.98 (0.92-0.98), indicating high consistency of heart position between simulation positioning and actual RT implementation. The ratio of heart D_mean_ of CBCT/DIBH-planned varied from 96.9% to 172.4%, with a median of 105% (Figure 4A).The heart D_mean_ of patients under VDIBH both in CBCT and DIBH-planned was significantly lower than that under FB (*P* < .005) (Figure 4B).

### Treatment Time and Acceptance

**Figure 5.**
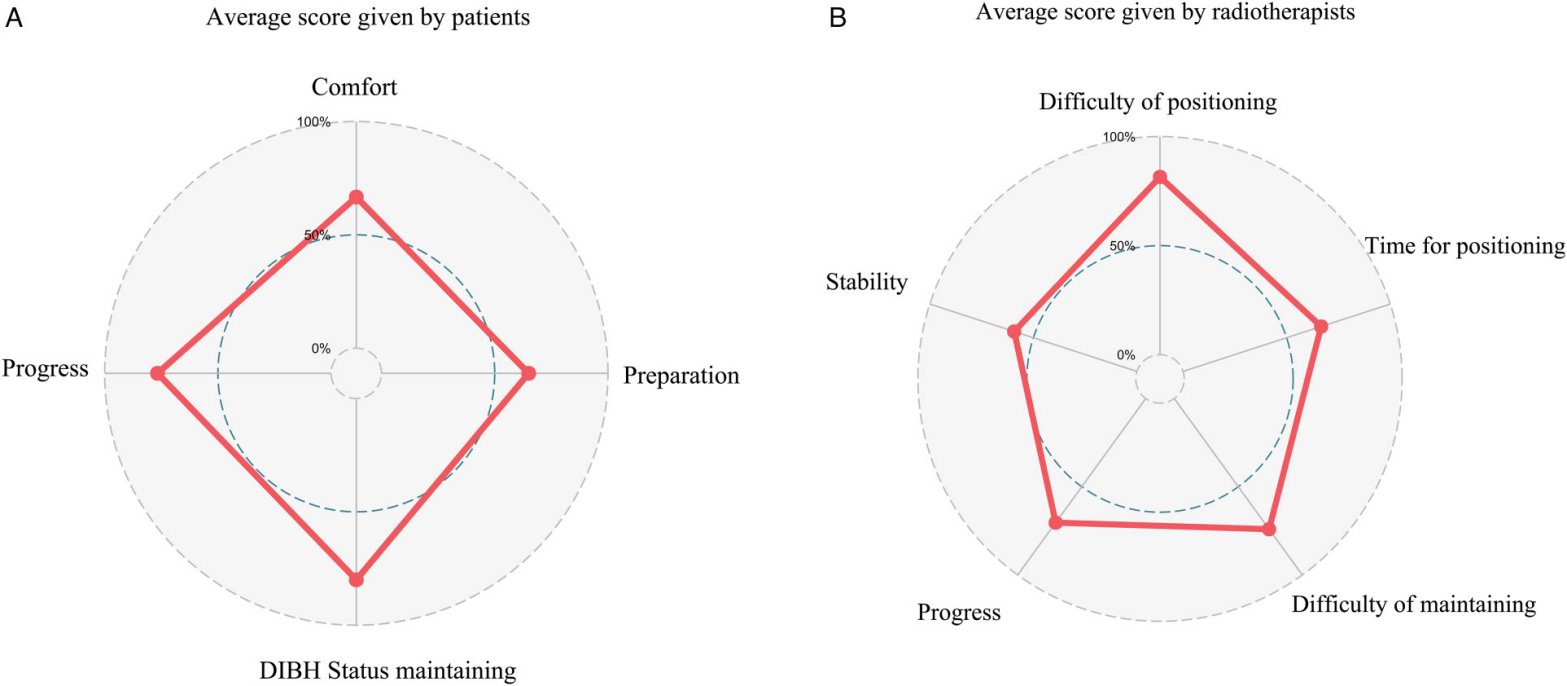
The average score for the acceptance of optical surface monitoring system (OSMS)-assisted voluntary deep inspiration breath-hold (VDIBH) treatment by patients (A) and radiotherapists (B).

The median treatment time of a single fraction was 4 (1.5-11) min. The median number of breath hold repetition per single treatment was 4 (2-9) times. The questionnaires were completed by all 20 patients with a median age of 45 years (range: 27-63) and 9 therapists with a median age of 37 years (range: 25-57) and median working years of 12 (range: 1-38). The average score for the acceptance of VDIBH by patients and therapists were 8.7 ± 0.9 (out of 12) and 10.6 ± 3.2 (out of 15), respectively, indicating good acceptance of the technique by both patients and therapists (Figure 5).

### PTV Margins

The median translational set up errors in X (lateral), Y (cephalo-caudal), and Z (ventral-dorsal) directions were 0.01 ± 0.14 cm, 0.02 ± 0.20 cm, and 0.06 ± 0.20 cm, respectively. The corresponding PTV margins were calculated as 0.49 cm, 0.69 cm, and 0.65 cm, respectively.

## Discussion

This study suggests that the cardiac radiation dose of left-sided breast cancer was significantly reduced by the use of VDIBH. The position of the heart was reproducible during treatment. The median treatment time of 4 min and feedback from patients and therapists indicated that RT using VDIBH and OSMS was acceptable. The PTV margins under VDIBH were <1 cm. In the present study, the heart D_mean_ was reduced by 55.3% and V20 of the left lung was significantly decreased, which is consistent with previous studies on DIBH (including ABC-DIBH and other DIBH).^6,8–10,18–22^

Previous studies on the effects of DIBH on cardiac radiation dose focused on the dosimetric analysis in treatment planning, which did not reflect the actual heart position and dose in daily treatment.^9,21^ In this study, we found high consistency of heart position and heart dose between daily CBCT and the planning CT.

Traditional ABC-DIBH devices have been widely used, and their physical benefits have been recognized by many studies. However, the process is complex, and many patients have difficulty accepting it. With the development and widespread use of the OSMS technology, the simpler VDIBH technology can be realized. We verified the accuracy and physical benefits of OSMS-assisted VDIBH. This is, to the best of our knowledge, the first study about the acceptance of OSMS-assisted VDIBH treatment by patients and therapists. A predesigned questionnaire was completed by patients and involved therapists to assess the acceptance of this technique. During the initial positioning, we let all patients experience ABC-DIBH training. With a full score of 15 points, 8 of 9 therapists scored more than 10 points, indicating good acceptance of VDIBH. But the oldest therapist (57 years) gave the lowest total score (3 points). No correlation between scores and age was found after statistical analysis. We also designed a question (No 4: How do you feel about VDIBH gating RT compared with utilization of ABC devices?) to compare the experience of ABC and VDIBH workflow. All patients gave 2 or 3 points, which means that VDIBH is more comfortable than ABC-DIBH.

In this study, the proposed PTV margins of 0.49 cm, 0.69 cm, and 0.65 cm in the lateral (L/R), cephalo-caudal (C/C), and ventral-dorsal (V/D) directions, respectively, were comparable with those under DIBH reported in literature (Table 2).

**Table 2.** The Corresponding PTV Margins of DIBH Reported in Studies (cm).

	X L/R		Y C/C		Z V/D		PTV margins		
	Σ	δ	Σ	δ	Σ	δ	X L/R	Y C/C	Z V/D
Bartlett *et al*^8^	0.44	0.38	0.49	0.33	0.33	0.26	1.37	1.46	1.01
Betgen *et al*^13^	0.22	0.2	0.5	0.35	0.33	0.27	0.69	1.50	1.01
Tanja *et al*^15^	0.17	0.13	0.15	0.15	0.15	0.12	0.52	0.48	0.46
Xiao *et al*^17^	N	N	N	N	N	N	0.61	0.47	0.49
Al-Hammadi *et al*^18^	0.13	0.04	0.21	0.07	0.15	0.06	0.35	0.57	0.42
This study	0.13	0.2	0.19	0.28	0.2	0.22	0.49	0.69	0.65

Abbreviation: DIBH, deep-inspiration breath hold.

In this study, VDIBH was thoroughly evaluated with respect to RT planning and technical implementation. The feasibility and physical advantages of the proposed therapy were clarified.^23^ Wang *et al* reported that the reduction of cardiac dose radiation is an independent prognostic factor for the incidence of subclinical cardiac events.^24^ Further research may prove that a reduction in cardiac radiation dose will result in a significant survival benefit for patients with breast cancer.

This study has limitations. First, in terms of OSMS reference images, no infrared camera was arranged at the CT simulation end, and the reference images of the surface could only be reconstructed based on the outer contour of the positioned CT images. Gierga *et al* demonstrated that the accuracy of the reference images reconstructed based on the outer contour was lower than that of the optical images directly collected at the CT simulation end.^25^ Second, the feasibility of VDIBH's physical advantages being transformed into long-term clinical cardiac benefits (cardiac indicators and the life quality) requires further long-term follow-up.

## Conclusions

Compared with FB, OSMS-assisted VDIBH for WBI can significantly reduce the cardiopulmonary radiation dose of patients with left-sided breast cancer. The heart position and dose in actual treatment were highly consistent with those in the planning system. Both patients and therapists exhibited good acceptance of the proposed therapy.

## Supplemental Material

sj-docx-1-tct-10.1177_15330338231173773 - Supplemental material for Voluntary Deep Inspiration Breath-Hold (VDIBH) Whole-Breast Irradiation Assisted by Optical Surface Monitoring System (OSMS) in Patients With Left-Sided Breast Cancer: A Prospective Phase II Study Click here for additional data file. Supplemental material, sj-docx-1-tct-10.1177_15330338231173773 for Voluntary Deep Inspiration Breath-Hold (VDIBH) Whole-Breast Irradiation Assisted by Optical Surface Monitoring System (OSMS) in Patients With Left-Sided Breast Cancer: A Prospective Phase II Study by Jiang-Hu Zhang, Tan-Tan Li, Shi-Rui Qin, Zhi-Qiang Liu, Si-Ye Chen, Yong-Wen Song, Yu Tang, Hao Jing, Hui Fang, Xu-Ran Zhao, Jing Jin, Yue-Ping Liu, Yuan Tang, Shu-Nan Qi, Ning Li, Bo Chen, Ning-Ning Lu, Ye-Xiong Li and Shu-Lian Wang in Technology in Cancer Research & Treatment

## Abbreviations

BCS: breast-conserving surgery
RT: radiotherapy
FB: free-breathing
DIBH: deep-inspiration breath hold
CBCT: cone beam computed tomography
OSMS: optical surface monitoring system
VDIBH: voluntary deep inspiration breath-hold
WBI: whole breast irradiation
LADCA: left anterior descending coronary artery
ROI: region of interest
ABC: active breathing control.
