# Supplementary material for: Voluntary Deep Inspiration Breath-Hold (VDIBH) Whole-Breast Irradiation Assisted by Optical Surface Monitoring System (OSMS) in Patients With Left-Sided Breast Cancer: A Prospective Phase II Study
Source: Technol Cancer Res Treat. 2023 Jun 14;22:15330338231173773. doi: 10.1177/15330338231173773 (PMC10285586; doi:10.1177/15330338231173773)
Supplement: sj-docx-1-tct-10.1177_15330338231173773 - Supplemental material for Voluntary Deep Inspiration Breath-Hold (VDIBH) Whole-Breast Irradiation Assisted by Optical Surface Monitoring System (OSMS) in Patients With Left-Sided Breast Cancer: A Prospective Phase II Study [file sj-docx-1-tct-10.1177_15330338231173773.docx]

**Appendix**

**Questionnaire for acceptance of VDIBH by patients**

|  | 0  Highly uncomfortable | 1  Uncomfortable | 2  Comfortable | 3  Highly comfortable |
| --- | --- | --- | --- | --- |
| 1. How do you feel about VDIBH gating radiotherapy? |  |  |  |  |
| 2. Do you feel comfortable about setup and staying in the correction position before treatment? |  |  |  |  |
| 3. Do you feel comfortable about entering and staying in the treatment status during treatment? |  |  |  |  |
| 4. How do you feel about VDIBH gating radiotherapy compared with utilization of ABC devices? |  |  |  |  |

**Questionnaire for acceptance of VDIBH by radiotherapists**

|  | 0  Highly agree | 1  Agree | 2  Sort of | 3  Disagree |
| --- | --- | --- | --- | --- |
| 1. The patient had difficulty in setup before treatment. |  |  |  |  |
| 2. Setup before treatment is time-consuming. |  |  |  |  |
| 3. It is difficult to enter and stay in the correction position during treatment. |  |  |  |  |
| 4. VDIBH gating radiotherapy is complicated/challenging compared with utilization of auxiliary devices. |  |  |  |  |
| 5. Treatment is frequently terminated as OSMS exceeds the threshold |  |  |  |  |
